# The complete mitochondrial genome of the scab mite *Psoroptes cuniculi* (Arthropoda: Arachnida) provides insights into Acari phylogeny

**DOI:** 10.1186/1756-3305-7-340

**Published:** 2014-07-22

**Authors:** Xiao-Bin Gu, Guo-Hua Liu, Hui-Qun Song, Tian-Yu Liu, Guang-You Yang, Xing-Quan Zhu

**Affiliations:** 1 State Key Laboratory of Veterinary Etiological Biology, Key Laboratory of Veterinary Parasitology of Gansu Province, Lanzhou Veterinary Research Institute, Chinese Academy of Agricultural Sciences, Lanzhou, Gansu Province 730046, P R China; 2 Department of Parasitology, College of Veterinary Medicine, Sichuan Agricultural University, Ya’an, Sichuan Province 625014, P R China

**Keywords:** *Psoroptes cuniculi*, Mitochondrial genome, Mitochondrial DNA, Phylogenetic analyses

## Abstract

**Background:**

Limited available sequence information has greatly impeded population genetics, phylogenetics and systematics studies in the subclass Acari (mites and ticks). Mitochondrial (mt) DNA is well known to provide genetic markers for investigations in these areas, but complete mt genomic data have been lacking for many Acari species. Herein, we present the complete mt genome of the scab mite *Psoroptes cuniculi*.

**Methods:**

*P. cuniculi* was collected from a naturally infected New Zealand white rabbit from China and identified by morphological criteria. The complete mt genome of *P. cuniculi* was amplified by PCR and then sequenced. The relationships of this scab mite with selected members of the Acari were assessed by phylogenetic analysis of concatenated amino acid sequence datasets by Bayesian inference (BI), maximum likelihood (ML) and maximum parsimony (MP).

**Results:**

This mt genome (14,247 bp) is circular and consists of 37 genes, including 13 genes for proteins, 22 genes for tRNA, 2 genes for rRNA. The gene arrangement in mt genome of *P. cuniculi* is the same as those of *Dermatophagoides farinae* (Pyroglyphidae) and *Aleuroglyphus ovatus* (Acaridae), but distinct from those of *Steganacarus magnus* (Steganacaridae) and *Panonychus citri* (Tetranychidae). Phylogenetic analyses using concatenated amino acid sequences of 12 protein-coding genes, with three different computational algorithms (BI, ML and MP), showed the division of subclass Acari into two superorders, supported the monophylies of the both superorders Parasitiformes and Acariformes; and the three orders Ixodida and Mesostigmata and Astigmata, but rejected the monophyly of the order Prostigmata.

**Conclusions:**

The mt genome of *P. cuniculi* represents the first mt genome of any member of the family Psoroptidae. Analysis of mt genome sequences in the present study has provided new insights into the phylogenetic relationships among several major lineages of Acari species.

## Background

The metazoan mitochondrial (mt) genome possess a circular double strand DNA that varies in size from 15 to 20 kb, generally encoding for 36–37 genes: 12–13 protein-coding genes (PCGs), two ribosomal RNAs (rRNA) genes and 22 transfer RNAs (tRNA) [1,2]. Mt genomes have been extensively used as genetic markers in molecular phylogenetic studies due to it having several useful properties (i.e., haploidy, compactness, maternal inheritance, relatively high mutation rates, and the lack of recombination) [3]. To date, there are over 4000 complete mt genome sequences of metazoans available in GenBank, including some from parasites [4-10]. In spite of the availability of advanced DNA technologies, however, there is still major gap in our knowledge of mt genomes of the subclass Acari.

Currently, the Acari encompasses over 55,000 species (ticks and mites) and forms the largest group among the class Arachnida [11]. Many species of ticks and mites threaten the health of plants, animals and humans on a global scale, causing major economic losses and significant public health problems [12,13]. Despite their abundance, medical, veterinary and economic significance, the genetics, epidemiology, biology and phylogenetic relationships in this group remain poorly understood. Acari were traditionally classified into three monophyletic lineages: superorders Parasitiformes (=Anactinotrichida), Opiloacariformes and Acariformes (=Actinotrichida) [14]. However, phylogenetic relationships among Acari have been revised in the past using nuclear rDNA gene sequences, indicating Opiloacariformes + Parasitiformes as the second monophyletic lineage of Acari [15,16]. Recently, mt genome sequences have been used to understand the phylogenetic relationships among Acari. Dermauw *et al.*[17] inferred the Acari phylogeny with the mt genome sequences of 21 species. Liu *et al.*[8] and Burger *et al.*[4] inferred the phylogeny of ticks with the mt genome sequences of 16 and 21 species, respectively. A difference among mt genome phylogenies is on the relationship within the order Ixodida. Dermauw *et al*. [17] and Burger *et al*. [4] showed strong support that Metastriate + Prostriate are sister to Ornithodorinae. However, Liu *et al.*[8] showed strong support that Ornithodorinae is more closely related to Prostriate, and Metastriate is sister to Ornithodorinae + Prostriate. More recently, Burger *et al.*[18] showed that Metastriate + Prostriate are sister to Ornithodorinae based on nuclear rDNA and mt gene sequences. The systematics and classification of the Acari are controversial in these analyses due to limited or underrepresented taxon sampling. So, the phylogenetic relationships inferred from nuclear and mt sequences need to be retested with more taxa from a wide range of lineages.

In the present study, we sequenced the complete mt genome of the scab mite *P. cuniculi* in the family Psoroptidae of the order Astigmata. *P. cuniculi* is a common worldwide parasite of rabbits, causing considerable economic loss due to weight loss, decreased food consumption, vestibular dysfunction, meningitis and death [19]. Also, we inferred the phylogenetic relationships with the concatenated mt amino acid sequences of *P. cuniculi* and 50 other Acari species that have been sequenced to date.

## Methods

### Ethics statement

This study was approved by the Animal Ethics Committee of The Lanzhou Veterinary Research Institute, Chinese Academy of Agricultural Sciences (Permit code. LVRIAEC2013-006). The New Zealand white rabbit, from which *P. cuniculi* was collected, was handled in accordance with good animal practices required by the Animal Ethics Procedures and Guidelines of the People’s Republic of China.

### Parasites and DNA extraction

*P. cuniculi* samples were collected from a naturally infected New Zealand white rabbit in Sichuan Province, China. The adult mites were isolated from ear cerumens of the rabbit, according to the previous descriptions [20]. The presence of mites was detected by light microscopic examination, and identification of *P. cuniculi* was conducted by morphological criteria and site of predilection [21], then fixed in 70% alcohol and stored at −20°C until use. Total genomic DNA was isolated from these specimens using sodium dodecyl sulphate/proteinase K treatment, followed by spin-column purification (Wizard^SV^ Genomic DNA Purification System, Promega). In brief, *P. cuniculi* collected from the same host were ground in a mortar and subsequently suspended in 275 μL lysis buffer (200 μL Nuclei Lysis Solution, 50 μL EDTA, 20 μL proteinase K and 5 μL RNase A Solution). After vortexing, the sample was incubated at 55°C for 16–18 h, and the DNA was extracted by shaking with 250 μL Wizard SV Lysis Buffer. After centrifugation, the DNA was washed, eluted and stored.

### PCR amplification and sequencing

Nine pairs of PCR primers were used to amplify overlapping segments of the complete mt genome of *P. cuniculi* as shown in Table 1. Briefly, the segment of *cox*1 was amplified using the primers COIF/COIR [22]. The other segments were amplified using designed primers (Table 1) based on sequences well conserved in the *Dermatophagoides farinae* and *D. pteronyssinus*[17,23]. All PCR reactions (25 μL) were performed in 2.5 mM dNTP mixture, 2.5 μL 10 × Ex *Taq* buffer (Mg^2+^ Plus), 10 pmol of each primer, 1 U Ex *Taq* polymerase (Takara), 1 μL of DNA sample in a thermocycler (Eppendorf, Germany) under the following conditions: 94°C for 5 min (initial denaturation); then followed by 35 cycles of 94°C for 30 s (denaturation), 36 ~ 60°C for 30 s (annealing), and 68°C for 1 ~ 4.5 min (extension) according to the product length; with a final extension step at 68°C for 10 min. Each PCR reaction yielded a single band as detected in a 1% (W/V) agarose gel upon ethidium-bromide staining (not shown). PCR products were subsequently sent to Invitrogen Biotechnology Company (Shanghai, China) for sequencing using a primer-walking strategy.

**Table 1:** **Sequences of primers used to amplify PCR fragments from *Psoroptes cuniculi***

**Primer**	**Sequence (5′ to 3′)**
COIF	GGTCAACAAATCATAAAGATATTGG
COIR	TAAACTTCAGGGTGACCAAAAAATC
COI-COIIF	TTAATTCTACATTCTTTGACC
COI-COIIR	GGAGAACTAGAATCTTGAAA
COII-ND3R	TTTCAAGATTCTAGTTCTCC
COII-ND3F	ACTCATAACARGTGGCTAAT
ND3-12SR	TTCTATTAGCCACCTGTT
ND3-12SF	AGTAACTCATTGGAAACC
12S-ND1R	AAACTAGGATTAGATACCA
12S-ND1F	ATTGCTTTTTTTACWTTAGT
ND1-ND4F	GCAGATAGGCTTATGACG
ND1-ND4R	AGAAAACAAAATAAAGGTAGG
ND4F	TGRCTTCCTAAAGCTCATG
ND4R	ATCTCAGAAAAAAAVGWTATAAAA
ND4-CytbF	TAGTGTTCATCTTTGACTTCC
ND4-CytbR	TACGCCATTTTACGGGCAATTCC
Cytb-COIF	CAGGGTTTGCTCCAATTCATGT
Cytb-COIR	CCCCTAAAATTGAAGAAATACCAGC

### Sequence analyses

Sequences were assembled manually and aligned against the complete mt genome sequences of *D. farinae* available using the computer program MAFFT 7.122 [24] to identify gene boundaries. Each gene was translated into amino acid sequence using the invertebrate mitochondrial genetic code in MEGA 5 [25], and aligned based on its amino acid sequence using default settings. The translation initiation and termination codons were identified to avoid gene overlap and to optimize the similarity between the gene lengths of closely-related mite mt genomes. For analyzing tRNA genes, the program tRNAscan-SE [26] and the program ARWEN [27] were used to detect tRNA and infer their secondary structure. For tRNAscan-SE, the following parameters were changed: Source: = Nematode Mito, Search Mode = tRNAscan > Cove, Genetic Code = Invertebrate Mito, Cove score cutoff = 0.1. EufindtRNA search parameters = relaxed. Putative secondary structures of 17 tRNA genes were identified, the remaining five tRNA genes (tRNA-Ser^UCN^, tRNA-Ile, tRNA-Pro, tRNA-Val and tRNA-Ala) were identified by recognizing potential secondary structures and anticodon sequences by eye, and two rRNA genes were predicted by comparison with those of other mites previously reported [17,23].

### Phylogenetic analyses

For comparative purposes, amino acid sequences predicted from published mt genomes of 50 Acari species were also included in the present analysis, using the scorpion *Centruroides limpidus* (GenBank accession number NC_006896) as an outgroup [28]. The 12 amino acid sequences (not ATP8) were single aligned using MAFFT 7.122 and then concatenated, and ambiguously aligned regions were excluded using Gblocks 0.91b (doc) with the default parameters [29] using the options for a less stringent selection. Phylogenetic analyses were conducted using three methods: Bayesian inference (BI), Maximum likelihood (ML) and Maximum parsimony (MP). The MtArt + I + G + F model of amino acid evolution was selected as the most suitable model of evolution by ProtTest 2.4 [30] based on the Akaike information criterion (AIC). As MtArt model is not implemented in the current version of MrBayes, an alternative model, MtREV, was used in BI and four chains (three heated and one cold) were run simultaneously for the Monte Carlo Markov Chain. Two independent runs for 1,000,000 metropolis-coupled MCMC generations, sampling a tree every 100 generation in MrBayes 3.1.1 [31]; the first 2,500 trees represented burn-in and the remaining trees were used to calculate Bayesian posterior probabilities (Bpp). The analysis was performed until the potential scale reduction factor approached 1 and the average standard deviation of split frequencies was less than 0.01. ML analysis was performed with PhyML 3.0 [32] using the subtree pruning and regrafting (SPR) method with a BioNJ starting tree, and the MtArt model of amino acid substitution with proportion of invariant sites (I) and gamma distribution (G) parameters estimated from the data with four discretized substitution rate classes, the middle of which was estimated using the median. Bootstrap frequency (Bf) was calculated using 100 bootstrap replicates. MP analysis was conducted using PAUP 4.0 Beta 10 program [33], with indels treated as missing character states; 1,000 random additional searches were performed using TBR. Bf was calculated using 1,000 bootstrap replicates, and 100 random taxon additions in PAUP. Phylograms were drawn using the program FigTree v.1.4.

## Results

### General features of the mt genome of *P. cuniculi*

**Figure 1:**
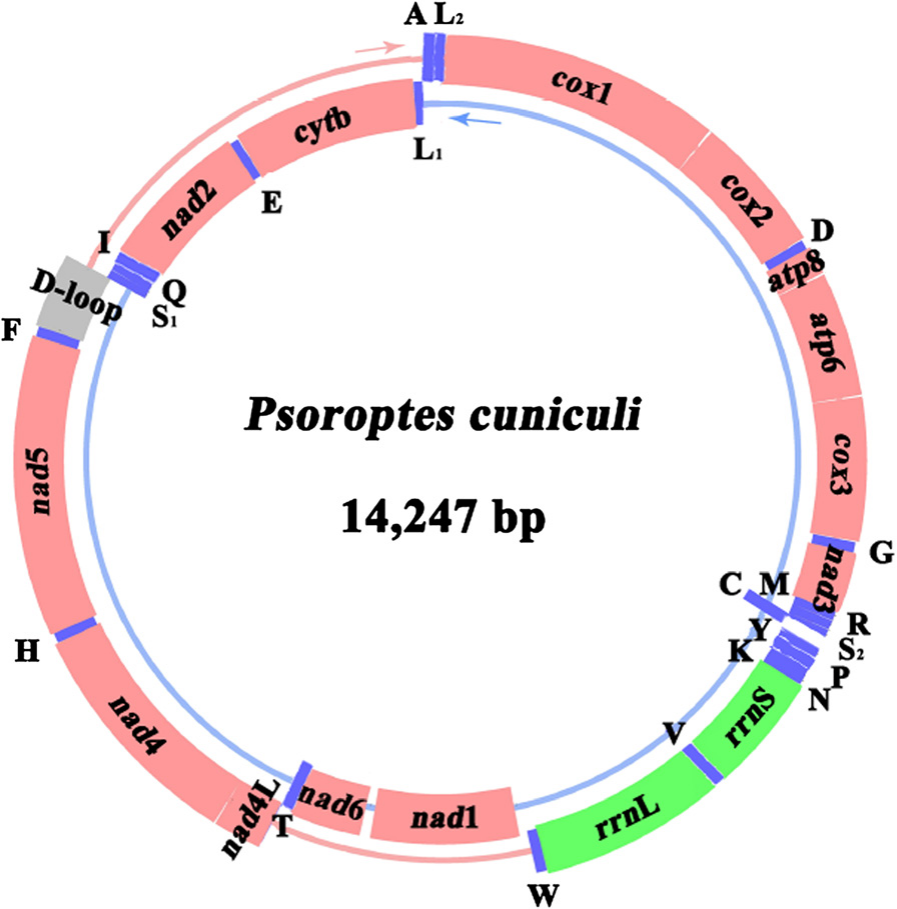
**Arrangement of the mitochondrial genome of *Psoroptes cuniculi*.** Gene scaling is only approximate. All genes have standard nomenclature including the 22 tRNA genes, which are designated by the one-letter code for the corresponding amino acid, with numerals differentiating each of the two leucine- and serine-specifying tRNAs (L_1_ and L_2_ for codon families CUN and UUR, respectively; S_1_ and S_2_ for codon families AGN and UCN, respectively).

The complete mt genome of *P. cuniculi* was 14,247 bp in length (Figure 1). This size is within the range of other mite mt genomes (Table 2). The sequences have been deposited in GenBank under the accession number KJ957822. This circular mt genome contains 13 PCGs (*cox*1-3, *nad*1-6, *nad*4L, *cyt*b, *atp*6 and *atp*8), 22 tRNA genes, two rRNA genes and one D-loop region (Table 3). The genes are transcribed in both different directions. Except for four PCGs (*nad*1, *nad*6, *nad*2 and *cyt*b) and seven tRNA genes (tRNA-Cys, tRNA-Thr, tRNA-Ser^AGN^, tRNA-Gln, tRNA- Ile, tRNA-Glu and tRNA-Leu^CUN^) encoded on the minority strand (N-strand), all other genes were encoded on the majority strand (J-strand) (Figure 1). The gene arrangement is the same as those of *D. farinae* (Pyroglyphidae) and *Aleuroglyphus ovatus* (Acaridae), but distinct from those of *Steganacarus magnus* (Steganacaridae) and *Panonychus citri* (Tetranychidae). The nucleotide composition of the complete mt genome of *P. cuniculi* is A = 29.4%, T = 41.7%, G = 16.8% and C = 12.1%, with a typically high A + T content of 71.1% within the range of values found in *D. farinae* (71.4%) and *D. pteronyssinus* (72.6%) (Table 2).

**Table 2:** Composition of mitochondrial genomes in the superorder Acariformes

**Species**	**Size (bp)**	**Accession number**	**A%**	**T%**	**G%**	**C%**	**A + T%**
*Aleuroglyphus ovatus*	14328	NC_023778	23.6	44.7	18.7	13.0	68.3
*Ascoschoengastia* sp.	16067	NC_010596	35.6	34.5	14.2	15.7	70.1
*Dermatophagoides farinae*	14266	NC_013184	26.7	44.7	17.6	11.0	71.4
*Dermatophagoides pteronyssinus*	14203	NC_012218	29.1	43.5	16.4	11.0	72.6
*Leptotrombidium akamushi*	13698	NC_007601	33.2	34.3	15.0	17.5	67.5
*Leptotrombidium deliense*	13731	NC_007600	34.4	35.6	14.1	15.9	70.0
*Leptotrombidium pallidum*	16779	NC_007177	34.4	36.6	13.9	15.1	71.0
*Panonychus citri*	13075	NC_014347	39.5	46.0	7.5	7.0	85.5
*Panonychus ulmi*	13115	NC_012571	40.3	45.3	7.2	7.2	85.6
***Psoroptes cuniculi***	**14247**	**KJ957822**	**29.4**	**41.7**	**16.8**	**12.1**	**71.1**
*Steganacarus magnus*	13818	NC_011574	36.5	38.1	12.2	13.2	74.6
*Tetranychus cinnabarinus*	13092	NC_014399	43.5	40.9	7.6	8.0	84.4
*Tetranychus urticae*	13103	NC_010526	43.2	41.1	7.7	8.0	84.3
*Unionicola foili*	14738	NC_011036	43.8	29.2	9.8	17.3	73.0
*Unionicola parkeri*	14734	NC_014683	46.6	26.2	9.9	17.3	72.8
*Walchia hayashii*	14857	NC_010595	46.1	26.9	9.4	17.6	73.0

**Table 3:** **Organization of *Psoroptes cuniculi *** **mitochondrial genome**

**Genes/regions**	**Position**	**Size (bp)**	**Strand**	**Start codons**	**Stop codons**
*cox*1	1-1548	1548	J	ATA	TAA
*cox*2	1562-2330	769	J	ATG	T
tRNA- Asp (D)	2332-2385	54	J		
*atp*8	2397-2543	147	J	ATA	TAG
*atp*6	2550-3221	672	J	ATG	TAA
*cox*3	3228-4010	783	J	ATG	TAA
tRNA-Gly (G)	4014-4069	56	J		
*nad*3	4075-4393	319	J	ATA	T
tRNA-Arg (R)	4395-4449	55	J		
tRNA-Met (M)	4454-4505	52	J		
tRNA-Ser^UCN^ (S2)	4506-4557	52	J		
tRNA-Cys (C)	4560-4609	50	N		
tRNA-Pro (P)	4619-4674	56	J		
tRNA-Tyr (Y)	4675-4728	54	J		
tRNA-Lys (K)	4719-4780	62	J		
tRNA-Asn (N)	4780-4837	58	J		
*rrn*S	4838-5495	658	J		
tRNA-Val (V)	5496-5547	52	J		
*rrn*L	5548-6571	1024	J		
tRNA-Trp (W)	6572-6628	57	J		
*nad*1	6690-7613	924	N	ATT	TAA
*nad*6	7625-8071	447	N	ATA	TAG
tRNA-Thr (T)	8074-8129	56	N		
*nad*4L	8136-8405	270	J	ATG	TAA
*nad*4	8406-9699	1294	J	ATG	T
tRNA-His (H)	9700-9754	55	J		
*nad*5	9775-11392	1618	J	ATT	T
tRNA-Phe (F)	11393-11446	54	J		
D-loop	11447-11828	382	J		
tRNA-Ser^AGN^ (S1)	11829-11881	53	N		
tRNA-Gln (Q)	11880-11931	52	N		
tRNA- Ile (I)	11935-11987	53	N		
*nad*2	12012-12912	901	N	TTG	T
tRNA-Glu (E)	12918-12970	53	N		
*cyt*b	12971-14070	1100	N	ATG	TA
tRNA-Leu^CUN^ (L1)	14080-14135	56	N		
tRNA-Ala (A)	14140-14189	50	J		
tRNA-Leu^UUR^ (L_2_)	14189-14245	57	J		

### Annotation

**Figure 2:**
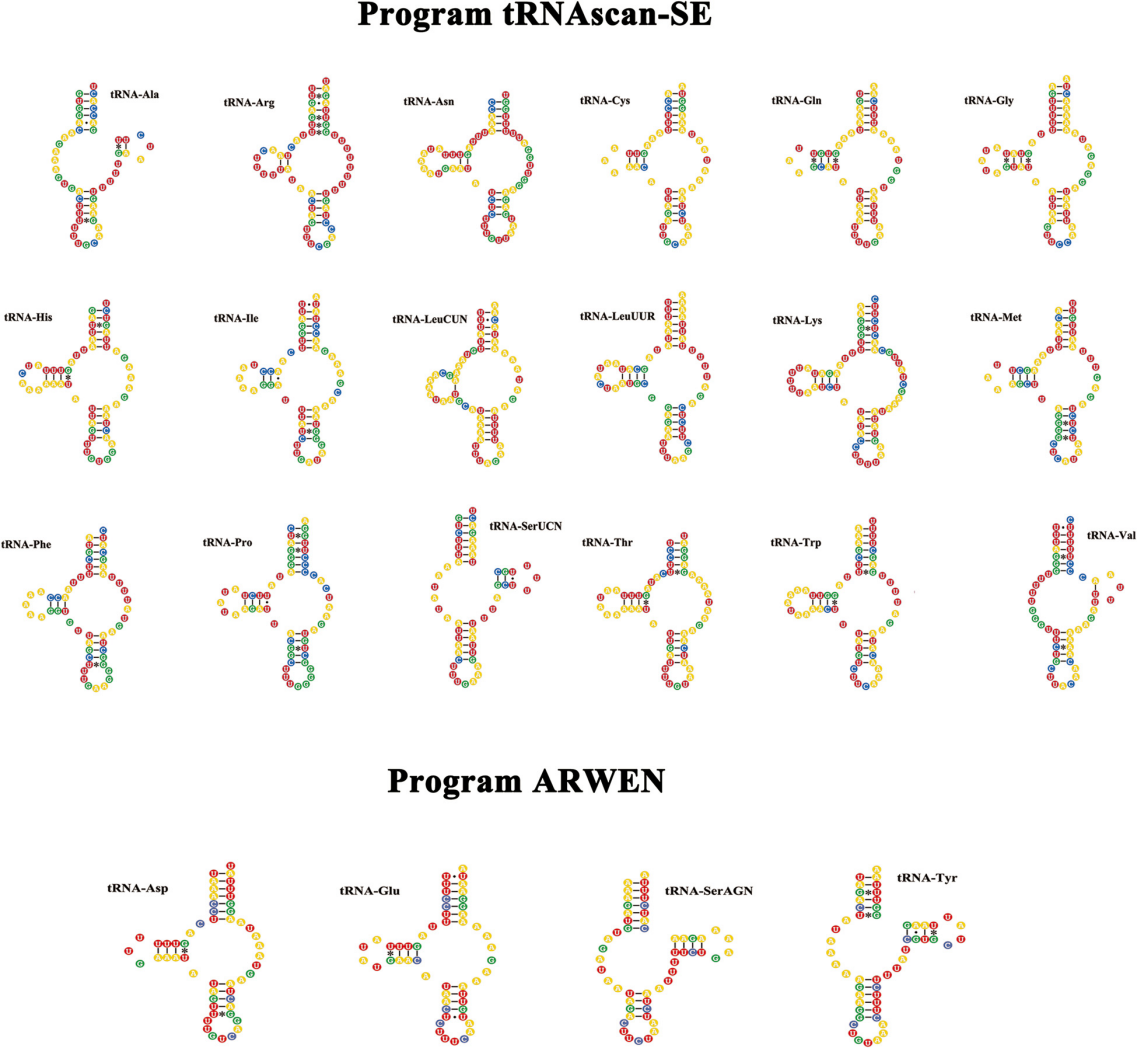
**Predicted secondary structures of the 22 mitochondrial tRNA of *Psoroptes cuniculi*.**

The length of PCGs of *P. cuniculi* was in the following order: *nad*5 > *cox*1 > *nad*4 > *cyt*b > *nad*1 > *nad*2 > *cox*3 > *cox*2 > *atp*6 > *nad*6 > *nad*3 > *nad*4L > *atp*8 (Table 3). A total of 3588 amino acids are encoded in the mt genome of *P. cuniculi*. In this mt genome, 4 genes (*cox*1, *nad*3, *atp*8 and *nad*6) use ATA, 6 genes (*cox*2, *cox*3, *atp*6, *nad*4, *nad*4L and *cyt*b) use ATG, 2 genes (*nad*1, *nad*5) use ATT and 1 gene (*nad*2) uses TTG as start codon, respectively (Table 3). All genes have complete termination codon except for *cyt*b, *cox*2, *nad*3, *nad*5, *nad*2 and *nad*4 genes which use abbreviated stop codon T or TA, 5 genes (*cox*1, *cox*3, *nad*1, *nad*4L and *atp*6) use TAA and 2 genes (*atp*8 and *nad*6) use TAG as termination codon, respectively (Table 3). The *rrn*S of *P. cuniculi* is located between tRNA-Asn and tRNA-Val, and *rrn*L is located between tRNA-Val and tRNA-Trp. The length of the *rrn*S gene is 658 bp and *rrn*L gene is 1024 bp (Table 3). The A + T contents of the *rrn*S and *rrn*L are 72.4% and 72.2%, respectively. As the mt genomes of Acariform mites can contain short minimal tRNA genes [23], the identification of tRNA genes can sometimes be challenging. A total of 22 tRNA sequences were identified in the *P. cuniculi* mt genome, ranging from 50–62 bp (Table 3). Almost all tRNA genes are atypical lacking either the T- or D-loop. Their predicted secondary structures (Figure 2) are similar to that of *D. farinae* and *D. pteronyssinus*. As in typical arthropod mtDNA, there is only a small D-loop region between tRNA genes. The D-loop of the *P. cuniculi* mt genome is located between the tRNA-Phe and tRNA-Ser^AGN^ (Table 3). The size of D-loop is 382 bp, and the A + T content is 89.8%. This region contains several dinucleotide [AT]_5–40_ repeats, which are conserved sequences in mt genomes of *D. farinae* and *D. pteronyssinus*[17,23]. In addition, this region holds a short palindromic sequence, TACAT, which is usually a conserved motif that is often found in mt genomes of insects, including *D. farinae* and *D. pteronyssinus*[17,23].

### Phylogenetic analyses

**Figure 3:**
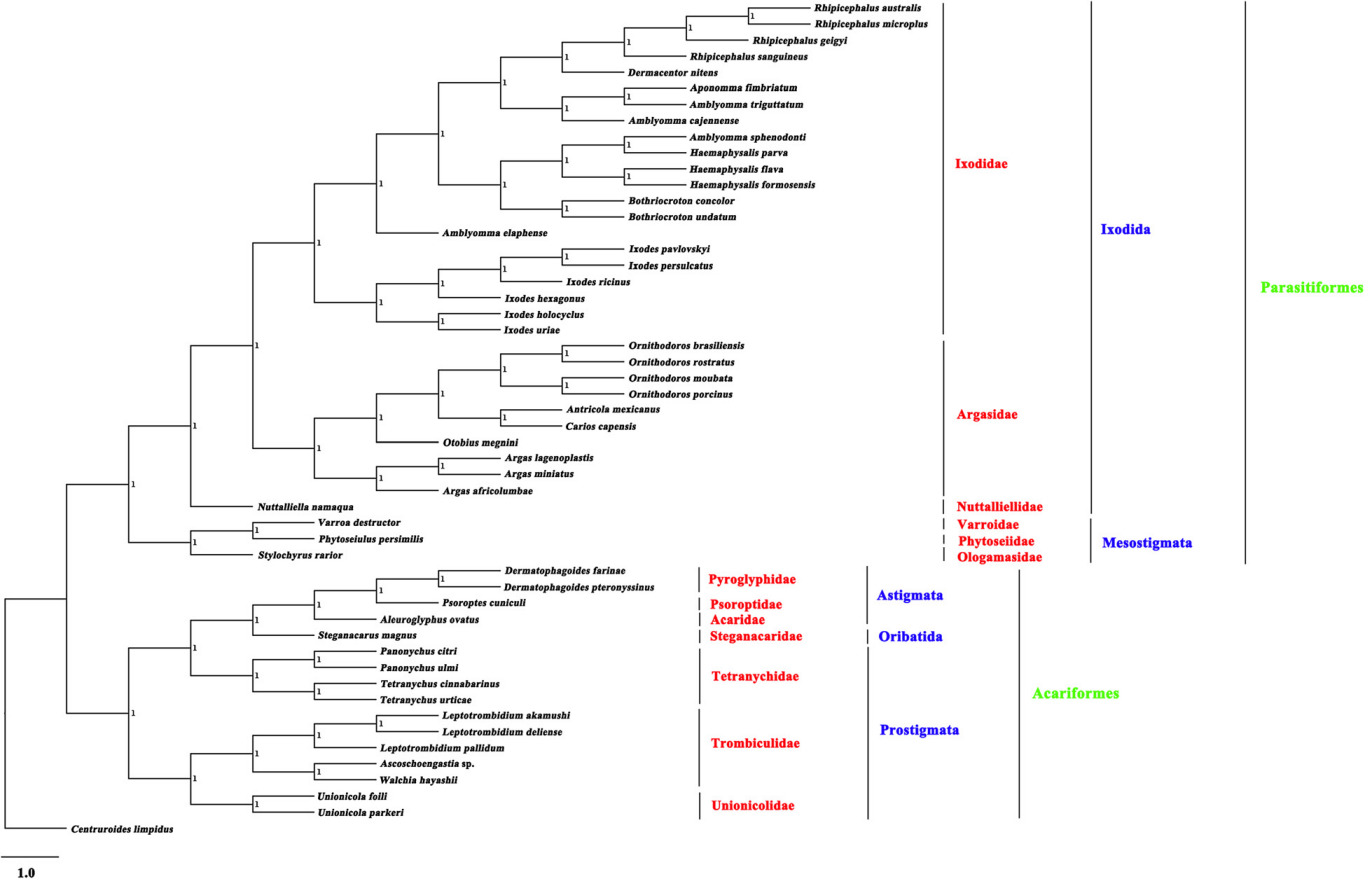
**Phylogenetic relationships among 51 species of Acari inferred from Bayesian analysis of deduced amino acid sequences of 12 mitochondrial proteins.***Centruroides limpidus* (GenBank accession number NC_006896) was used as the outgroup. Bayesian posterior probability (Bpp) values were indicated at nodes.

**Figure 4:**
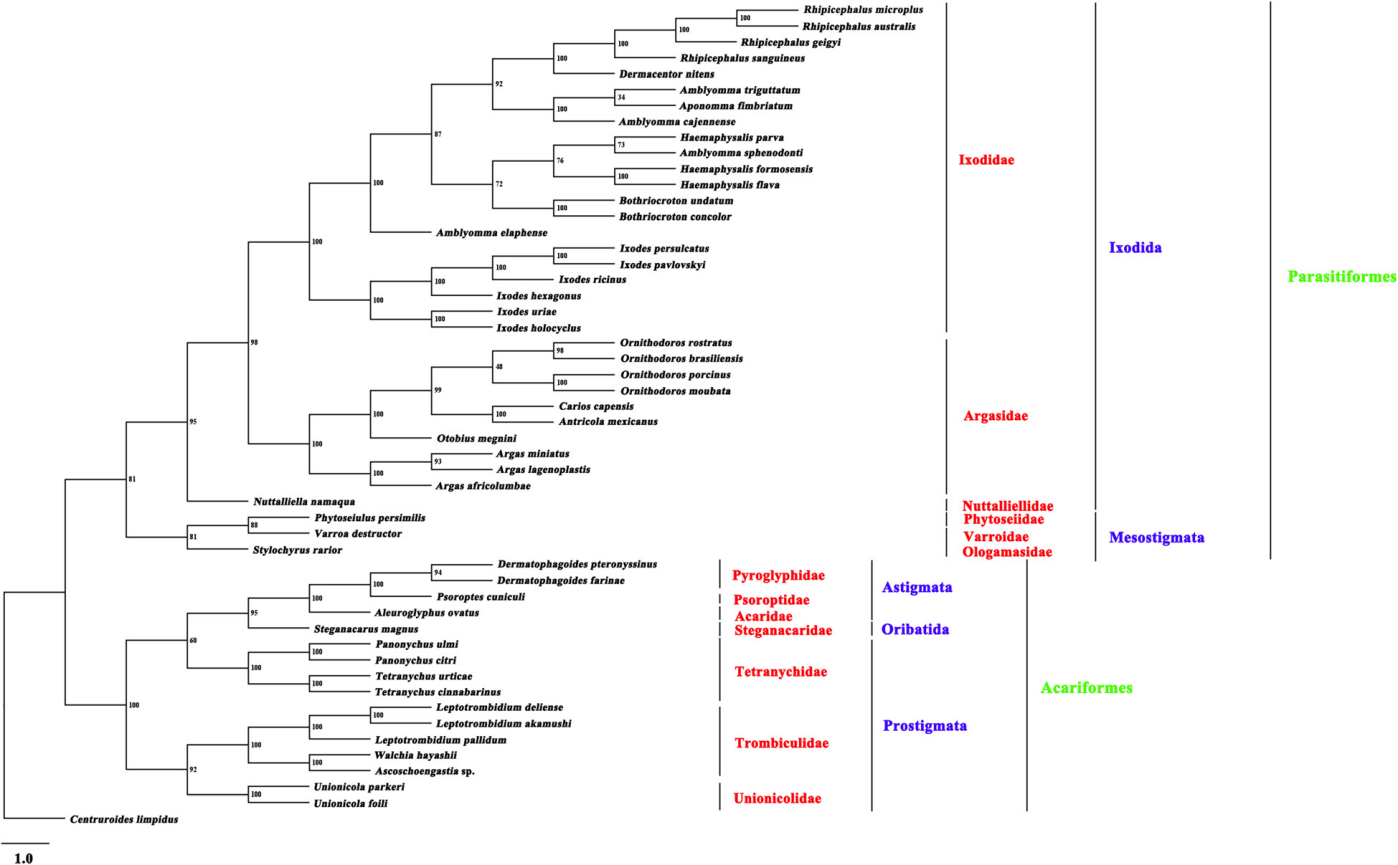
**Phylogenetic relationships among 51 species of Acari inferred from maximum likelihood (ML) of deduced amino acid sequences of 12 mitochondrial proteins.***Centruroides limpidus* (GenBank accession number NC_006896) was used as the outgroup. Bootstrapping frequency (Bf) values were indicated at nodes.

**Figure 5:**
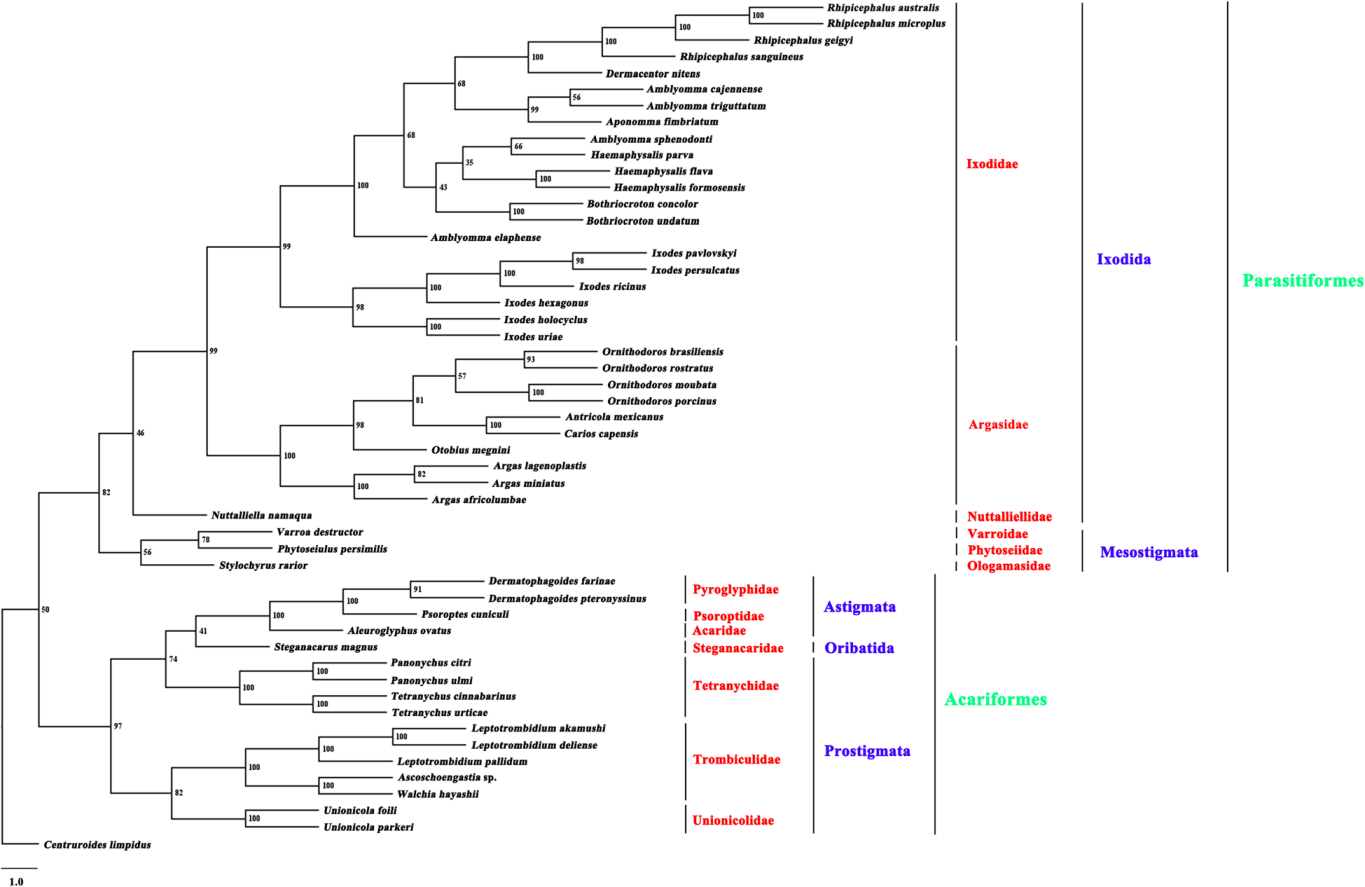
**Phylogenetic relationships among 51 species of Acari inferred from maximum parsimony (MP) of deduced amino acid sequences of 12 mitochondrial proteins.***Centruroides limpidus* (GenBank accession number NC_006896) was used as the outgroup. Bootstrapping frequency (Bf) values were indicated at nodes.

Of the 51 Acari species included in the phylogenetic analyses in this study, 35 species belonged to the superorder Parasitiformes while 16 belonged to the superorder Acariformes. Both superorders Parasitiformes and Acariformes were monophyletic in all of the trees inferred by the BI, ML and MP methods. The monophyly of the superorder Parasitiformes was strongly supported with a Bpp of 1 in BI analyses (Figure 3), a Bf of 81% in ML analyses (Figure 4), and a Bf of 82% in MP analyses (Figure 5). The monophyly of the superorder Acariformes was strongly supported in BI and ML analyses (Bpp = 1, Figure 3; Bf = 100%, Figure 4), and was moderately supported in MP analysis (Bf = 97%, Figure 5). The 35 species of ticks in the superorder Parasitiformes included in this study were from two orders: Ixodida (32 species) and Mesostigmata (3 species). The monophyly of the order Ixodida was strongly supported in BI and ML analyses (Bpp = 1, Figure 3; Bf = 95%, Figure 4), but was weakly supported in MP analysis (Bf = 46%, Figure 5). The Mesostigmata were monophyletic with strong support in BI analysis (Bpp = 1, Figure 3), and was moderately supported in ML analysis (Bf = 81%, Figure 4), and weakly supported in MP analysis (Bf = 56%, Figure 5). Of the 16 species of mites in the superorder Acariformes, 4 species were from the order Astigmata, one species from the order Oribatida and 11 species from the order Prostigmata. The Astigmata was monophyletic with strong support in all of the three phylogenetic analyses (Bpp = 1, Figure 3; Bf = 100%, Figure 4; Bf = 100%, Figure 5). However, the Prostigmata was not monophyletic in all of the three phylogenetic analyses in the present study (Figures 3, 4 and 5). The Prostigmata was paraphyletic with respect to the family Tetranychidae. Four species from the Tetranychidae of the Prostigmata were more closely related to families Steganacaridae (order Oribatida), Acaridae, Psoroptidae and Pyroglyphidae (order Astigmata) than they were to the other two families (Trombiculidae and Unionicolidae) of the order Prostigmata. The close relationship between these species of the Tetranychidae and Steganacaridae was strongly supported in BI analysis (Bpp = 1, Figure 3), and was moderately supported in ML and MP analyses (Bf = 60%, Figure 4; Bf = 74%, Figure 5). In addition to the Prostigmata, four families of the orders Astigmata and Oribatida were also represented in our analyses: Pyroglyphidae (2 species), Psoroptidae (1 species), Acaridae (1 species) and Steganacaridae (1 species). The Pyroglyphidae was monophyletic with strong support in all of the three phylogenetic analyses in this study (Bpp = 1, Figure 3; Bf = 94%, Figure 4; Bf = 91%, Figure 5). Of the 32 species of ticks in the Ixodida, 21 species were from the family Ixodidae (hard ticks), 10 species from the family Argasidae (soft ticks) and one species from the family Nuttalliellidae. The two families Ixodidae and Argasidae were monophyletic with strong support in BI analysis (Bpp = 1, Figure 3), ML analysis (Bf = 100%, Figure 4) and MP analysis (Bf > 99%, Figure 5). The four species of the genus *Amblyomma* within Ixodidae were included in this study. These results indicated that *Amblyomma* is paraphyletic with strong support in BI analysis (Figure 3), and is moderately supported in ML and MP analyses (Figures 3 and 4).

## Discussion

For two decades, there has been considerable debate surrounding the systematics of Acari. The subclass Acari were initially recognized as three lineages (Acariformes, Parasitiformes and Opiloacariformes), however, some authors later demonstrated that there are two distinct groups: Acariformes and Parasitiformes [34-37], and Opiloacariformes as an independent lineage were rejected as they represent basal Parasitiformes [14]. Unfortunately, as there are no complete mt genomes of Opilioacariformes available, we were not able to verify this hypothesis. Dabert *et al*. [38] confirmed the monophyly of Acariformes based on the nuclear small subunit rRNA gene (18S rDNA) and the mt cytochrome c oxidase subunit I (COX1). Dermauw *et al.*[17] tested the hypothesis of the Acari phylogeny with the mt genome sequences of 21 species, and showed strong support of the monophylies of the superorders Parasitiformes and Acariformes, represented by 12 species from the Parasitiformes and 9 species from the Acariformes. This study, however, could not reject the monophyly of the order Prostigmata. In the present study, our analyses support the division of the subclass Acari into two superorders Parasitiformes and Acariformes [14], however, the order Prostigmata was paraphyletic in all of the three phylogenetic analyses in this study. Although morphological characteristics remain an important source of phylogenetic signal in organismal evolution, morphological characters and molecular characters vary among orders and major groups of Acari. Prostigmata phylogeny based on morphological characteristics shows that the order Prostigmata has an apparent monophyly and is sister to orders Endeostigmata + Oribatids [39]. However, molecular data provide some support that the order Prostigmata was paraphyletic in the current study. However, we should be cautious that the phylogenetic signal may be influenced by long-branch attraction artifacts, this phenomenon may challenge the analyses of phylogeny of Acariform mites [38].

The superorder Parasitiformes is composed of four orders: Opilioacarida, Holothyrida, Ixodida, and Mesostigmata, and the superorder Acariformes is divided into two suborders: Sarcoptiformes (consisting of Endeostigmata, Oribatida and Astigmata) and Trombidiformes (Prostigmata or Actinedida) [40]. Domes *et al.* (2007) showed that the order Astigmata is located on the outside of the order Oribatida, usually as sister-group of the order Endeostigmata [41]. However, Dabert *et al.*[38] speculated that order Oribatida is paraphyletic with respect to order Astigmata. In the present study, although our results have shown that the orders Oribatida and Astigmata are sister groups, only one species within the order Oribatida was included in the present study.

In the present study, our results provide strong support that families Ixodidae and Argasidae are sister taxa, consistent with those of previous studies [4,42]. Here, we will not discuss the phylogenetic relationships of other families within the superorder Parasitiformes (eg., Nuttalliellidae, Phytoseiidae, Varroidae and Ologamasidae) due to limited data. Phylogenetic analyses based on mt genome sequences in the present study strongly support a close relationship between families Tetranychidae and Steganacaridae + Acaridae + Psoroptidae + Pyroglyphidae to the exclusion of families Trombiculidae and Unionicolidae. Apparently, additional markers for phylogenetic inference are required to resolve the controversy on the order Prostigmata between phylogeny based on mt genome sequences and nuclear SSU rRNA sequences [15]. The monophyly of the order Oribatida will also need further testing with expanded taxon sampling as only one species, *Steganacarus magnus*, from the Steganacaridae was included in the present study. No species from the superorder Opilioacariformes and order Holothyrida was included in our analyses. Therefore, expanding taxon sampling from these lineages of Acari is clearly the next step for phylogenetic studies of Acari using mtDNA. In addition, phylogenetic analysis in the present study was based only on mtDNA sequences, so we believe it is still necessary to employ nuclear genomic sequences to provide additional evidence for phylogenetic analyses and genome evolution of the Acari.

## Conclusion

The present study determined the mt genome of the mite species *P. cuniculi* of animal health significance, and represents the first mt genome study of any member of the family Psoroptidae. Phylogenetic analyses of the mt genome sequences of the *P. cuniculi* species, in combination with 50 other Acari species, support the monophylies of the superorders Parasitiformes and Acariformes; the orders Ixodida, Mesostigmata and Astigmata, however, reject the monophyly of the order Prostigmata. Our results indicate the need to test hypotheses of Acari phylogeny with more taxa and different molecular markers.

## Competing interests

The authors declare that they have no competing interests.

## Authors’ contributions

XBG, GHL, HQS, TYL and GYY performed the experiments, analyzed the data and drafted parts of the manuscript. XQZ conceived and funded the study, revised and edited the manuscript. All authors read and approved the final manuscript.
